# Seroconversion of SSA and the Development of Inflammatory Myositis in a Patient With Chronic Joint Pain: A Potential Overlap Connective Tissue Disease

**DOI:** 10.7759/cureus.91619

**Published:** 2025-09-04

**Authors:** Diva Maraj, Iman El-Feki, Hazem Zebda

**Affiliations:** 1 Internal Medicine, Henry Ford Health System, Jackson, USA; 2 Rheumatology, Henry Ford Health System, Detroit, USA

**Keywords:** anti-ssa, connective tissue disease, inflammatory myositis, mixed connective tissue disease, myositis

## Abstract

We present the case of a 48-year-old female with a complex medical history, including chronic joint pain on methadone and chronic obstructive pulmonary disease (COPD), who developed right forearm myositis following a series of orthopedic procedures and infections. Initially thought to be infectious in origin, subsequent investigations revealed chronic inflammation without infection. The patient’s serologic profile evolved over time, with new-onset SSA antibody positivity, positive antinuclear antibody (ANA) and rheumatoid factor (RF), and concern for overlapping connective tissue disease. This case highlights the diagnostic complexity and evolving nature of autoimmune conditions, particularly in patients with non-specific musculoskeletal symptoms and comorbidities.

## Introduction

Connective tissue diseases often present with vague and overlapping symptoms, which may evolve over time. Sjögren syndrome, systemic lupus erythematosus (SLE), and inflammatory myopathies, such as polymyositis, can co-exist or mimic each other [1]. Early recognition and serological monitoring are critical, especially in patients with persistent joint symptoms and systemic findings [2,3]. This case describes a patient with longstanding joint pain, initially managed as osteoarthritis, who later demonstrated features consistent with autoimmune diseases, including inflammatory myositis and SSA seroconversion. SSA antibodies are relevant, as they provide more information about other autoimmune diseases patients may have, including SLE, Sjogren's, and mixed connective tissue diseases, which can guide therapy [2,3].

## Case presentation

A 48-year-old female, with a history of chronic joint pain managed with maintenance methadone therapy and chronic obstructive pulmonary disease (COPD), presented with right forearm swelling in 2025. This followed recent lower extremity hardware removal and prior foot surgery complicated by infection requiring incision and drainage (I&D) and six weeks of intravenous antibiotics.

**Figure 1:**
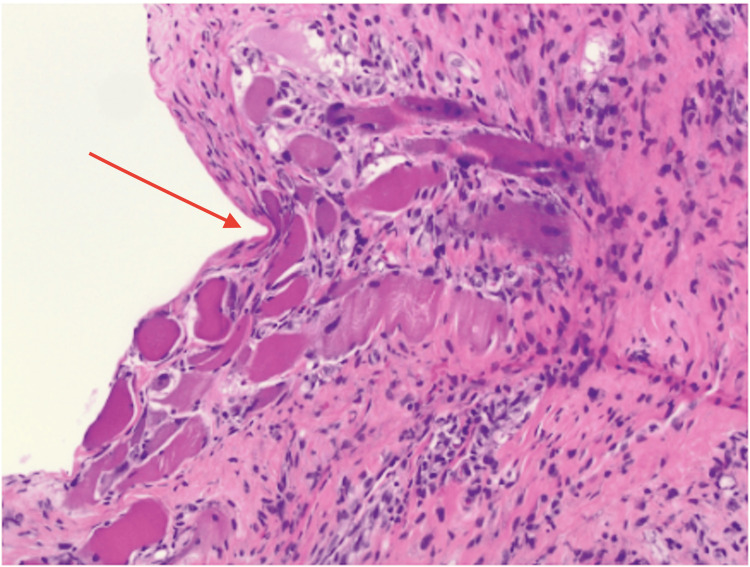
Myositis biopsy, with hematoxylin and eosin staining at 100 amplification, showing chronic inflammation

**Figure 2:**
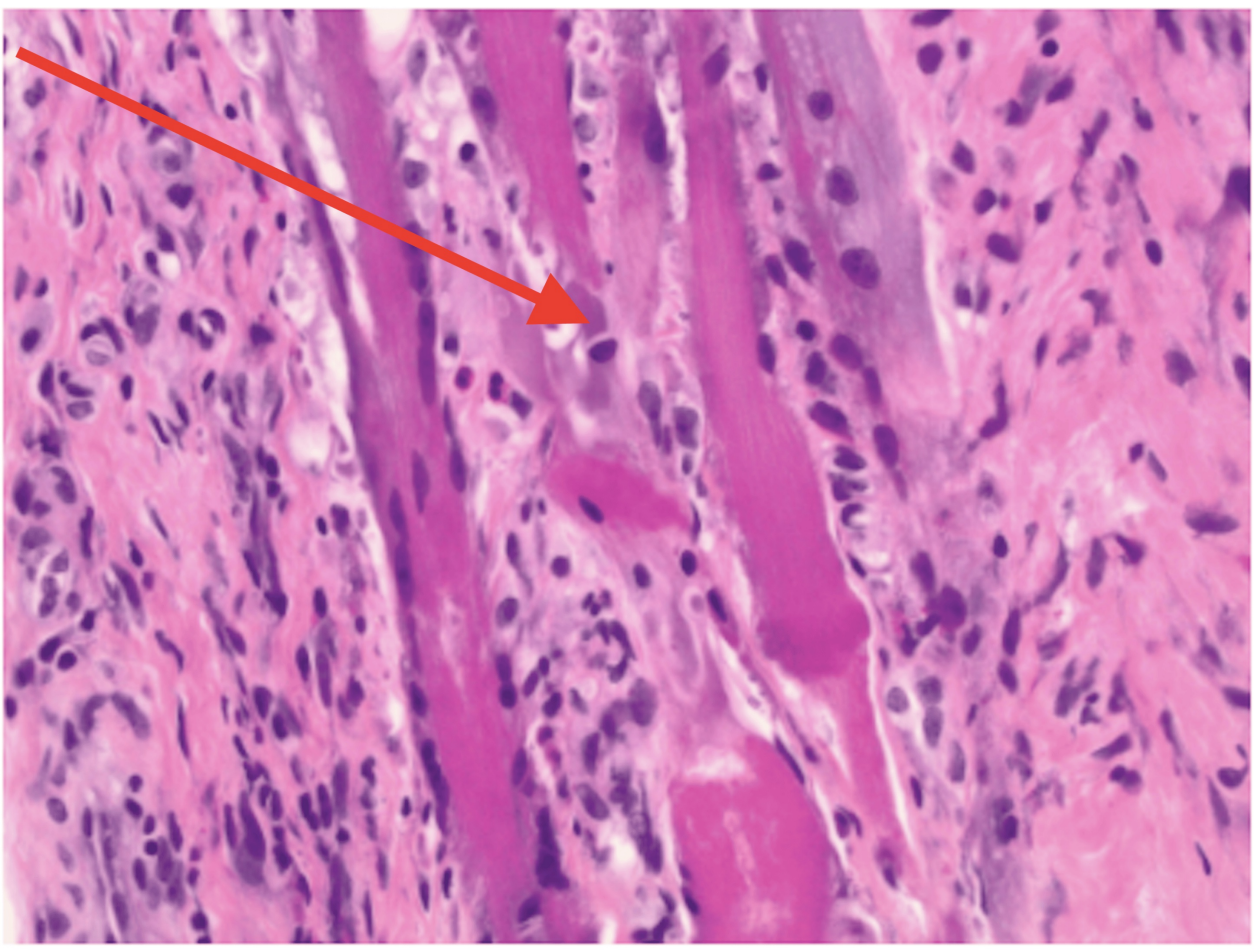
Fibrosis seen on biopsy, with hematoxylin and eosin staining at 100 magnification

Initial evaluation raised concern for infectious myositis such as overlap myositis or polymyositis. She underwent surgical I&D and biopsy, which revealed chronic inflammation and fibrosis without microbial growth (Figure 1 and Figure 2).

A comprehensive myositis panel was negative at that time. The myositis panel included testing for anti-Jo-1 and SRP, which were negative (Table 1). She was treated empirically with intravenous Ceftriaxone and Daptomycin per infectious disease recommendations.

**Table 1:** Autoimmune workup, initial presentation

Test	Reference Range	Result	Comment
RNP Antibody	< 1.0 ELISA Units	< 0.2	Negative
SM Antibody	< 1.0 ELISA Units	< 0.2	Negative
SS-A/Ro Antibody	< 1.0 ELISA Units	0.3	Negative
SS-B/La Antibody	< 1.0 ELISA Units	< 0.2	Negative
ANA (Antinuclear Ab)	—	1:640	Positive, Speckled Pattern
RF (Rheumatoid Factor)	< 14 IU/mL (Nephlometry)	18	High

The patient had been experiencing chronic joint pain for several years, involving the bilateral hands, knees, and feet. She reported persistent stiffness lasting all day. Previously diagnosed with osteoarthritis, she had undergone multiple orthopedic evaluations and foot surgery in July 2022. A planned total knee arthroplasty in 2023 was canceled due to failed pulmonary clearance in the context of her COPD.

She had a history of positive rheumatoid factor (RF) and antinuclear antibody (ANA), although prior SSA antibodies were negative in 2023. She denied other systemic symptoms, including rash, photosensitivity, oral ulcers, Raynaud’s, alopecia, or constitutional symptoms. There was no personal or family history of psoriasis, inflammatory bowel disease, or uveitis.

Despite multiple referrals, the patient had inconsistent follow-up with rheumatology until her first completed visit in April 2022. In 2025, following the forearm myositis episode, a repeat autoimmune workup revealed SSA antibody seroconversion. Repeat serology also showed continued ANA and RF positivity, with a positive ANA titer of 1:1280 and a speckled pattern. AVISE (Exagen Inc., Vista, CA, US) testing confirmed strong ANA and RF reactivity, though overall interpretation remained indeterminate (Tables 2-4). AVISE testing refers to a specialized set of blood tests that are used in the diagnosis and management of autoimmune diseases, particularly SLE and related connective tissue diseases with overlapping symptoms.

**Table 2:** Positive ANA serology ANA: antinuclear antibody

Test	Result	Interpretation
ANA by HEp-2 (IFA)	Titer: 1:1280	Positive
Nuclear Pattern	Speckled	—
Cytoplasmic Pattern	Not Observed	—

**Table 3:** Lupus score biomarkers Net MFI: mean fluorescence intensity

Test	Value	Interpretation	Reference Range
Anti-dsDNA IgG (ELISA)	32.45 IU/mL	Negative	<201 - Negative | 201–302 - Equivocal | >302 - Positive
Anti-Smith IgG (ELFA)	<0.7 U/mL	Negative	<7 - Negative | 7–10 - Equivocal | >10 - Positive
CB-CAP: EC4d	11 Net MFI	Negative	<15 - Negative | 15–75 - Positive | >75 - Strong Positive
CB-CAP: BC4d	12 Net MFI	Negative	<61 - Negative | 61–200 - Positive | >200 - Strong Positive

**Table 4:** ANA testing ANA: antinuclear antibody

Test	Result	Interpretation	Reference Range
ANA IgG	60.93 Units	Strong Positive	<20 = Negative, 20–60 = Positive, >60 = Strong Positive
Anti-SSB/La IgG	<0.4 U/mL	Negative	<7 = Negative, 7–10 = Equivocal, >10 = Positive
Anti-Scl-70 IgG	<0.6 U/mL	Negative	<7 = Negative, 7–10 = Equivocal, >10 = Positive
Anti-CENP-B IgG	<0.4 U/mL	Negative	<7 = Negative, 7–10 = Equivocal, >10 = Positive
Anti-Jo-1 IgG	<0.3 U/mL	Negative	<7 = Negative, 7–10 = Equivocal, >10 = Positive
Anti-CCP IgG	0.9 U/mL	Negative	<7 = Negative, 7–10 = Equivocal, >10 = Positive

Inflammatory markers were mildly elevated, with ESR 44 mm/hr and CRP 3 mg/L. Hepatitis B and C testing were negative.

Given the development of biopsy-confirmed inflammatory myositis and new serological findings, there was increased concern for an evolving connective tissue disease.

Potential differential diagnoses included Sjogren syndrome overlapping with SLE or inflammatory arthritis overlapping with inflammatory myopathy, or undifferentiated connective tissue disease, due to the pattern of symptoms and incomplete serologic specificity. The patient was started on methotrexate 15 mg weekly and folic acid supplementation for suspected inflammatory arthritis/myositis overlap, with improvement in symptoms.

## Discussion

This case illustrates the progression from presumed mechanical joint disease to an inflammatory autoimmune process in a patient with long-standing symptoms. Notably, the evolution of serological markers, particularly SSA seroconversion, raises concern for a systemic connective tissue disease. SSA antibody positivity is a hallmark of Sjogren syndrome and may also be seen in SLE and myositis [3,4].

This case is unusual in that the patient’s trajectory began with features suggestive of a mechanical or degenerative joint process, only later evolving into a picture more consistent with systemic autoimmunity. Such a shift underscores the heterogeneity of autoimmune disease presentations and the potential for long latency periods before classical serologies become positive. The emergence of SSA seroconversion after years of symptoms is particularly notable, as it is uncommon for patients with longstanding presumed non-inflammatory joint disease to later declare a systemic autoimmune condition [3-6].

The association of SSA positivity with both inflammatory myositis and connective tissue disease further complicates the clinical picture. While SSA antibodies are most often linked to Sjögren’s syndrome, they can also signal overlap syndromes, such as SLE-myositis or Sjögren-myositis, which are relatively rare [4,5,7]. The coexistence of inflammatory arthritis, biopsy-proven chronic inflammation, and SSA seropositivity raises the possibility of an evolving overlap syndrome, an entity that is diagnostically challenging but clinically important, given its therapeutic implications.

While initial concern centered on infection, the lack of microbial growth on culture and chronic inflammation on the biopsy shifted the differential toward an autoimmune pathology. The AVISE test is an autoimmune workup, which tests for all autoimmune conditions, such as Lupus, Sjogren's syndrome, inflammatory myositis, and mixed connective tissue diseases that present with overlapping symptoms [8-11]. The delay in diagnosis highlights the importance of ongoing surveillance and re-evaluation, especially when initial serologic tests are unrevealing [8,9].

The overlap of inflammatory myositis and systemic autoimmune diseases poses diagnostic challenges [5]. Methotrexate initiation is appropriate for inflammatory arthritis and has efficacy in some myositis subtypes [7,8,10]. Continued monitoring and potential escalation of immunosuppressive therapy may be warranted based on clinical response.

## Conclusions

This case underscores the importance of serial evaluation and serologic testing in patients with chronic joint pain and systemic symptoms. SSA seroconversion and inflammatory myositis in this patient raise the possibility of overlapping autoimmune syndromes, warranting close monitoring and immunomodulatory therapy.
